# Partnerships for detecting emerging infectious diseases: Nepal and global influenza surveillance.

**DOI:** 10.3201/eid0401.980122

**Published:** 1998

**Authors:** J M Gambel, D R Shlim, L C Canas, N J Cox, H L Regnery, R M Scott, D W Vaughn, C H Hoke, P W Kelley


Because *C. cayetanensis* is a seasonal diarrheal agent, fecal samples from persons with persistent unexplained explosive diarrhea during the summer should be carefully evaluated for this infection. Stool specimens should be fixed in 10% formalin and examined with autofluorescence microscopy for enhanced detection.

**O.G.W. Berlin,*† J.B. Peter,† C. Gagne,† C.N. Conteas,‡ and L.R. Ash***
*School of Public Health, University of California, Los Angeles, California, USA; †Specialty Laboratories, Santa Monica, California, USA; and ‡Kaiser Permanente Medical Center, Los Angeles, California, USA

### References

1. Berlin OGW, Novak SM, Porschen RK, Long EG, Stelma GN, Schaeffer FW. Recovery of *Cyclospora* organisms from patients with prolonged diarrhea. Clin Infect Dis 1994;18:606-9.
2. Ash LR, Orihel TC. Collection and preservation of feces. Parasites: a guide to laboratory procedures and identification. Chicago: ASCP Press; 1991. p. 3-53.
3. Weber R, Bryan RT, Owen RL, Wilcox CM, Gorelkin L, Visvesvara GS. Improved light microscopical detection of microsporidia spores in stool and duodenal samples. N Engl J Med 1992;326:161-6.
4. Berlin OGW. Mycobacteria. In: Baron EJ, Finegold SM, editors. Diagnostic microbiology. 8th ed. St. Louis: C.V. Mosby; 1990. p. 597-640.
5. Ash LR, Orihel TC. Atlas of human parasitology. 4th ed. Chicago: ASCP Press; 1997.
6. Centers for Disease Control and Prevention. Update: outbreaks of cyclosporiasis—United States, 1997. MMWR Morb Mortal Wkly Rep 1997;46:451-2.
7. Centers for Disease Control and Prevention. Update: outbreaks of cyclosporiasis—United States and Canada, 1997. MMWR Morb Mortal Wkly Rep 1997;46:521-3.
8. Colley DG. Widespread food-borne cyclosporiasis outbreaks present major challenges. Emerg Infect Dis 1996;2:354-6.
9. Eberhard ML, Pienazek NJ, Arrowood MJ. Laboratory diagnosis of *Cyclospora* infections. Arch Pathol Lab Med 1997;121:792-7.

### Partnerships for Detecting Emerging Infectious Diseases: Nepal and Global Influenza Surveillance

**To the Editor:** With new influenza strains emerging each year, identification of circulating strains by coordinated global surveillance is crucial to vaccine development for the coming year (1-3). Approximately 110 laboratories in 80 countries voluntarily participate in the World Health Organization (WHO) influenza surveillance network (4). Comprehensive surveillance is especially important in Asia, since new influenza strains often originate there. To participate in influenza global surveillance, countries need not rely on their own laboratory capability. Clinical specimens from patients thought to have influenza can be sent to designated laboratories around the world for analysis. A unique partnership has led to the expansion of the WHO global influenza surveillance network to Nepal.

The U.S. Army Medical Component - Armed Forces Research Institute for Medical Sciences (AFRIMS) (5) in Bangkok, Thailand, is well situated to assist with surveillance in Asia. Scientists at AFRIMS have conducted medical research in collaboration with Nepali colleagues for more than 20 years. Several studies have been conducted in collaboration with the CIWEC Clinic Travel Medicine Center (a travel medicine clinic that serves the diplomatic, aid, and tourist communities in Nepal). The clinic has approximately 5,000 patient visits per year, of which half are drawn from the 2,500 expatriates in Nepal and half from the 200,000 non-Indian tourists who visit Nepal annually.

A protocol was developed for a pilot influenza surveillance program. The staff of the CIWEC Clinic was responsible for volunteer recruitment, clinical evaluation, and specimen collection. Febrile upper respiratory infections were defined as temperature ≥ 100°F (37.8°C, oral or equivalent) and cough or sore throat of ≤ 72 hours duration. Other symptoms, such as streptococcal pharyngitis, were excluded. No age or gender restrictions were included. Volunteers had to have been in Nepal for the 5 days preceding illness. Only the first patient in any single household with similar symptoms within days of other household members was asked to participate.

The AFRIMS field station in Kathmandu (locally known as the Walter Reed/AFRIMS Research Unit - Nepal or WARUN) was responsible for shipping specimens collected by the CIWEC Clinic to AFRIMS, Thailand. Since dry ice was not available in Kathmandu, dry ice and shipping containers were sent by AFRIMS, Thailand for use by WARUN. Shipments from WARUN were then sent back to AFRIMS, where specimens were repacked in dry ice and sent for testing at the central laboratory of the U.S. Air Force's Project Gargle (6) in San Antonio, Texas. Project Gargle has been testing viral respiratory specimens from distant Air Force installations for more than 20 years. Each specimen was tested for influenza A and B; parainfluenza virus 1, 2, and 3; adenovirus; enterovirus; and herpesvirus. Characterization of selected influenza A and B isolates by hemagglutination-inhibition testing was performed by the Centers for Disease Control and Prevention (CDC).

Between December 1996 and February 1997, the CIWEC staff collected specimens from 18 patients. Samples were collected from 11 (61%) residents and seven (39%) tourists, who were evenly distributed by gender and had a median age of 35 years. Influenza B/Beijing/184/93-like viruses were isolated from five (28%) of the 18 specimens. All patients from whom influenza viruses were obtained had mild illnesses with fever and upper respiratory syndromes. Herpes virus type 1 and adenovirus type 6 were each identified in one other specimen. No respiratory viruses were identified in the remaining 11 specimens.

Because of the importance of China in the emergence of new strains of influenza, CDC's WHO Collaborating Center for Surveillance, Epidemiology, and Control of Influenza has worked with colleagues in China to establish a national Chinese network of influenza surveillance sites. Analysis of viruses isolated in China between 1988 and 1997 in comparison with other viruses obtained through WHO's global influenza surveillance network has shown that influenza variants are frequently identified in China before becoming prevalent in other regions of the world. Nepal is another especially valuable surveillance site, given its location between China and India (at the crossroads between northern and southern Asia) and its historic importance as a trans-Himalayan trade route.

Especially relevant are data from China demonstrating that the two antigenically and genetically distinct lineages of influenza B viruses represented by B/Victoria/02/87 and B/Yamagata/16/88 (7) have continued to circulate and evolve in China, while only viruses related to B/Yamagata have been detected elsewhere in the world and are represented in the current trivalent vaccine by the B/Bejing/184/93-like component. Virologic surveillance in surrounding countries (8) such as Nepal is necessary to detect geographic spread of B/Victoria-like virus in the region. Our data suggest that these viruses have not yet spread to Kathmandu.

Our unique international partnership between several civilian and military organizations (e.g., CIWEC Clinic, CDC, U.S. Air Force, and U.S. Army) demonstrates the feasibility of such partnerships as well as the usefulness of influenza surveillance data at both the local and global levels. Despite the small number of isolates obtained during this study, we were able to determine that the influenza B component of the trivalent vaccine prepared for the 1996-1997 influenza season would likely have offered protection for travelers and the local population against the influenza B strains isolated in Kathmandu. Ongoing surveillance data will establish geographic and temporal patterns of circulation of influenza viruses and thus provide valuable information for guiding public health policies for influenza vaccination. On a global level, these data are useful for annual vaccine strain selection.

Advances in communication, laboratory, and specimen transport technologies contributed greatly to the identification of viral pathogens from a new sentinel surveillance site in Nepal. In evaluating future collaborative sites, prior surveillance experience and reliable specimen shipping should be prime considerations. Approaches that use existing resources might foster greater international cooperation toward improved global detection and reporting of infectious diseases.

### Acknowledgment

Financial support was given by the United States Department of Defense Emerging Infectious Disease Program.

**Jeffrey M. Gambel,* David R. Shlim,† Linda C. Canas,‡ Nancy J. Cox,¶ Helen L. Regnery,§ Robert M. Scott,§ David W. Vaughn,§ Charles H. Hoke Jr.,* and Patrick W. Kelley***

*Walter Reed Army Institute of Research, Washington, D.C., USA; †CIWEC Clinic, Kathmandu, Nepal; ‡Armstrong Laboratory - Diagnostic Virology, Brooks Air Force Base, Texas, USA; ¶Centers for Disease Control & Prevention, Atlanta, Georgia, USA; and §Armed Forces Research Institute of Medical Sciences, Bangkok, Thailand

### References

1. Gross PA. Preparing for the next influenza pandemic: a reemerging infection. Ann Internal Med 1996;124:682-5.

2. Glezen WP. Emerging infections: pandemic influenza. Epidemiol Rev 1996;18:64-76.
3. Patriarca PA, Cox NJ. Influenza pandemic preparedness plan for the United States. J Infect Dis 1997;176(Suppl 1):S4-S7.
4. Hampson AW. Surveillance for pandemic influenza. J Infect Dis 1997;176(Suppl 1):S8-S13.
5. Gambel JM, Hibbs RG. U.S. military overseas medical research laboratories. Mil Med 1996;161:638-45.
6. Williams RJ, Cox NJ, Regnery HL, Noah DL, Khan AS, Miller JM, ET AL. Meeting the challenge of emerging pathogens: the role of the United States Air Force in global influenza surveillance. Mil Med 1997;162:82-6.
7. Rota PA, Hemphill ML, Whistler T, Regnery HL, Kendal AP. Antigenic and genetic characterization of the hemagglutinins of recent circulating strains on influenza type B virus. J Gen Virol 1992:73:2737-42.
8. Centers for Disease Control and Prevention. Update influenza activity—United States and worldwide, 1996-97 season, and composition of the 1997-98 influenza vaccine. MMWR Morb Mortal Wkly Rep 1997;46:325-30.

### HIV-2 Infection and HIV-1/HIV-2 Dual Reactivity in Patients With and Without AIDS-Related Symptoms in Gabon

**To the Editor**: Between 1996 and 1997, we evaluated the incidence of HIV-2 infection at the Fondation Jeanne Ebori, the second largest hospital in Libreville, capital of Gabon; we found an unexpected high prevalence of HIV-2–infected or HIV-1/HIV-2–dually reactive patients.

During a 10-month period, 147 (14.3%) of 1,029 sera from inpatients and outpatients were found HIV-positive by the type III method recommended by the World Health Organization (two enzyme-linked immunosorbent assays are used to screen anti-HIV antibodies) (1). Further discrimination between HIV-1 and HIV-2 infections was assessed by using synthetic peptides specific for the gp41 and the gp120 of HIV-1 and the gp36 of HIV-2 (ImmunoComb II, PBS Orgenics, Illkirch, France). Of the 147 HIV-positive sera, 141 (96.0%) were exclusively HIV-1–positive; four were exclusively HIV-2–positive; and two were both HIV-1– and HIV-2–positive. Of the six sera with anti-gp36/HIV-2 reactivities, two (from patients A and B) were positive on HIV-2 Western blot, with marked anti-gag HIV-1 cross-reactivity and a discrimination assay positive only for HIV-2; two (from patients D and E) were positive on HIV-2 Western blot, with anti-gag and pol reactivities markedly lower than anti-env reactivities and a discrimination assay positive only for HIV-2; the two remaining sera (from patients C and F) showed typical dual reactivities for HIV-1 and HIV-2 infections, with positive patterns of HIV-1 and HIV-2 Western blots and a discrimination test positive for both viruses. As a whole, six (4.1%) of 147 HIV-positive sera showed either HIV-2 infection alone (n = 4) or dual reactivity. Of those, four were from Gabonese patients B, C, D, and E, and two were from immigrants from West Africa (patient A from Mali and patient F from Nigeria); two were female patients B and E. Among Gabonese patients, only one (patient E) had traveled to West Africa; the remaining three had never visited any neighboring country. However, one Gabonese man (patient C) lived in Port-Gentil, which has many West African immigrants. For all patients, the most likely risk factor for HIV was a heterosexual relationship with an unknown HIV-infected person. In three asymptomatic patients (A, B, and C) the HIV-2–serostatus was unexpected; in contrast, the three other patients had AIDS-related symptoms. Patients D and E had an HIV-2 Western blot pattern showing a marked decrease in anti-gag and pol reactivities compatible with their advanced stage of HIV-2 disease.

The case of a 55-year-old exclusively heterosexual asymptomatic woman (patient B) suggests the possibility of a specific variant of HIV-2 in Central Africa (2). The high frequency in primates in Gabon of natural infection with simian immunodeficiency retroviruses, which show a high degree of genetic relatedness to HIV-2 (3), could support such a hypothesis.

Two patients had typical dual reactivities to HIV-1 and HIV-2 antigens. To our knowledge, such dual reactivities have never been reported in Gabon (4). In the patient from Nigeria (patient F), the serologic pattern was typical of that usually observed in West Africa (5). Dual reactivity can result from genuine mixed infections and from serologic cross-reactivity in HIV-1 and HIV-2 infection alone; theoretically, it could also represent infection with a different, cross-reacting recombinant strain (5).

HIV-2 infection in Gabon is epidemiologically related to West Africa, because of cultural and, above all, economic ties. However, HIV-2 is not limited to immigrant populations from West Africa or to Gabonese citizens traveling in this area; it has also reached the indigenous Gabonese

